# Galectins in Intestinal Inflammation: Galectin-1 Expression Delineates Response to Treatment in Celiac Disease Patients

**DOI:** 10.3389/fimmu.2018.00379

**Published:** 2018-03-01

**Authors:** Victoria Sundblad, Amado A. Quintar, Luciano G. Morosi, Sonia I. Niveloni, Ana Cabanne, Edgardo Smecuol, Eduardo Mauriño, Karina V. Mariño, Julio C. Bai, Cristina A. Maldonado, Gabriel A. Rabinovich

**Affiliations:** ^1^Laboratorio de Inmunopatología, Instituto de Biología y Medicina Experimental (IBYME), Consejo Nacional de Investigaciones Científicas y Técnicas (CONICET), Buenos Aires, Argentina; ^2^Centro de Microscopía Electrónica, Facultad de Ciencias Médicas, Universidad Nacional de Córdoba, Córdoba, Argentina; ^3^Instituto de Investigaciones en Ciencias de la Salud (INICSA), Consejo Nacional de Investigaciones Científicas y Técnicas (CONICET), Córdoba, Argentina; ^4^Laboratorio de Glicómica Funcional y Molecular, Instituto de Biología y Medicina Experimental (IBYME), Consejo de Investigaciones Científicas y Técnicas (CONICET), Buenos Aires, Argentina; ^5^Sección Intestino Delgado, Departamento de Medicina, Hospital de Gastroenterología Dr. C. Bonorino Udaondo, Buenos Aires, Argentina; ^6^Unidad de Patología, Hospital de Gastroenterología, Bonorino Udaondo, Buenos Aires, Argentina; ^7^Instituto de Investigaciones, Universidad del Salvador, Buenos Aires, Argentina; ^8^Departamento de Química Biológica, Facultad de Ciencias Exactas y Naturales, Universidad de Buenos Aires, Buenos Aires, Argentina

**Keywords:** celiac disease, galectin-1, galectins, glycans, gut inflammation, inflammatory bowel disease

## Abstract

Galectins, a family of animal lectins characterized by their affinity for N-acetyllactosamine-enriched glycoconjugates, modulate several immune cell processes shaping the course of innate and adaptive immune responses. Through interaction with a wide range of glycosylated receptors bearing complex branched N-glycans and core 2-O-glycans, these endogenous lectins trigger distinct signaling programs thereby controling immune cell activation, differentiation, recruitment and survival. Given the unique features of mucosal inflammation and the differential expression of galectins throughout the gastrointestinal tract, we discuss here key findings on the role of galectins in intestinal inflammation, particularly Crohn’s disease, ulcerative colitis, and celiac disease (CeD) patients, as well as in murine models resembling these inflammatory conditions. In addition, we present new data highlighting the regulated expression of galectin-1 (Gal-1), a proto-type member of the galectin family, during intestinal inflammation in untreated and treated CeD patients. Our results unveil a substantial upregulation of Gal-1 accompanying the anti-inflammatory and tolerogenic response associated with gluten-free diet in CeD patients, suggesting a major role of this lectin in favoring resolution of inflammation and restoration of mucosal homeostasis. Thus, a coordinated network of galectins and their glycosylated ligands, exerting either anti-inflammatory or proinflammatory responses, may influence the interplay between intestinal epithelial cells and the highly specialized gut immune system in physiologic and pathologic settings.

## Introduction: Deciphering Glycocodes in Immunity

Complex sugar structures play essential roles as hardware for storage of biological information, which can be deciphered by endogenous glycan-binding proteins or lectins (1). The singular role of lectins in translating glycan-containing information into a myriad of cellular responses invigorated further studies aimed at understanding their expression patterns and molecular mechanisms of action.

Galectins, a family of lectins with affinity for N-acetyllactosamine (LacNac) residues, have diverse roles in shaping the course of innate and adaptive immunity and tailoring inflammatory responses, thereby modulating tumor immunity and autoimmune reactions (2, 3). In this perspective article, we review current knowledge on the role of galectins in inflammatory intestinal disorders, and present new findings on the regulated expression of galectin (Gal)-1 in intestinal tissue of celiac disease (CeD) patients.

## Galectins

Galectins, evolutionarily conserved glycan-binding proteins, play key roles in multiple immune cell processes. Either through protein-glycan or protein–protein interactions, these lectins function within the extracellular milieu by interacting with various glycosylated receptors, or work inside the cells by controlling distinct signaling pathways and modulating intracellular processes (3, 4).

To date, 15 members of the galectin family have been identified in vertebrates, which were classified into three groups based on their molecular architecture: (a) “proto-type” galectins, comprising a single polypeptide chain with one carbohydrate recognition domain (CRD) that is able to dimerize (Gal-1, -2, -5, -7, -10, -11, -13, -14, and -15); (b) “chimera-type” Gal-3, which consists of a C-terminal CRD linked to an N-terminal peptide, and (c) “tandem repeat-type” galectins composed of a single polypeptide chain exhibiting two CRDs in tandem connected by a linker peptide (Gal-4, -6, -8, -9, and -12) (4, 5). While some members of the family (e.g., Gal-1 and Gal-3) are widely distributed among different tissues and species (6–8), others have more restricted tissue localization. For example, Gal-7 is preferentially found in the skin (9, 10), Gal-12 is mostly expressed in adipose tissue (11, 12), Gal-5 is restricted to rat reticulocytes (13, 14) and Gal-10 is found in human but not mouse eosinophils (15).

Once synthesized, galectins may remain within the intracellular compartment and participate in protein-protein interactions to regulate intracellular events (16, 17). For example, both Gal-1 and -3 participate in pre-mRNA splicing (18) whereas Gal-10 modulates functionality of human CD25^+^ Treg cells (19). However, despite the lack of a classical secretory signal peptide, most galectins are released through an unconventional route to the extracellular compartment (20). Secreted galectins can specifically decipher biological information encoded in complex saccharide structures (particularly LacNac-enriched complex branched N-glycans and core 2 O-glycans), and convey this biochemical information into functional cellular responses (3, 17). Although saccharide structures are widely distributed in a range of glycoconjugates, individual galectins may co-opt a particular set of glycosylated receptors, generated by the coordinated action of glycosyltransferases and glycosidases which are differentially regulated in distinct target cells (4, 21, 22). Notably, one-CRD galectins can dimerize *via* the back sides of their CRDs, whereas chimera-type Gal-3 can pentamerize *via* its non-lectin N-terminal domain, and tandem-repeat galectins can oligomerize (17). Thus, through formation of multivalent galectin–glycan complexes, galectins can promote cross-linking, reorganization, and clustering of glycosylated receptors thereafter regulating their activation and signaling (23, 24). Within the immune compartment, galectin–glycan complexes may control signaling thresholds of relevant receptors such as the T-cell receptor (25), pre-B cell receptor (26), and cytokine receptors (27) among others, thereby modulating lymphoid and myeloid regulatory programs.

## Galectins: Key Players in the Inflammatory Response

Compelling evidence highlights major roles for galectins in controlling innate and adaptive immune responses. These lectins may influence the capacity of innate immune cells [e.g., neutrophils, dendritic cells (DCs), monocytes/macrophages, eosinophils, and mast cells] to respond to chemotactic gradients, migrate across endothelial cell surfaces, synthesize and release pro- or anti-inflammatory cytokines, and recognize, engulf, and kill microbes and damaged cells (28). In this regard, some galectins trigger innate immune responses, while others influence the resolution of acute inflammation (28). Galectins can also tailor adaptive immunity by influencing T-cell signaling and activation, modulating T-cell survival, controlling the suppressive function of regulatory T cells (Tregs), altering the cytokine balance and regulating B-cell maturation and differentiation (3). Both the specificity of the CRD as well as glycan presentation in the corresponding receptors make distinct contributions to the specific effects of individual galectins, selectively mediating different biological processes. The final balance of their synchronized actions contributes to activation, polarization, and resolution of adaptive immune responses (29). Although the specific immunoregulatory activities of each individual galectin is beyond the scope of the present work, and are described elsewhere (3, 29), some of the most relevant activities displayed by Gal-1, the central core of the present article, are summarized herein. This endogenous lectin, composed of two subunits of 14.5 kDa, functions as a regulatory signal which undermines acute inflammatory responses by controlling neutrophil adhesion, function and turnover (30, 31) and modulating monocyte and macrophage activation and polarization (32–35). Moreover, Gal-1 influences DC maturation, immunogenicity, and migration (36–40). Interestingly upon exposure to this lectin, DCs acquire an IL-27-dependent regulatory function leading to IL-10-mediated T-cell tolerance, suppression of T-helper (Th)1 and Th17 responses, promotion of tumor-immune escape and suppression of autoimmune neuroinflammation (40).

Regarding the T-cell compartment, Gal-1 controls T-cell viability, blunts Th1- and Th17-mediated responses and skews the balance of the immune response toward a Th2 cytokine profile (17, 41–43). Interestingly, we found that Th1- and Th17-differentiated cells express the repertoire of cell surface glycans that are critical for Gal-1 binding and induction of apoptosis; whereas Th2 cells are protected from this lectin through α2,6-sialylation of surface glycoproteins (43). Remarkably, Gal-1 also controls the immunosuppressive activity of Tregs and promotes their differentiation (44–46). Finally, by influencing B-cell development, differentiation, signaling and survival, Gal-1 also controls B-cell function (47–50).

The essential role of Gal-1 in the control of inflammation has been widely demonstrated in experimental models of autoimmunity, allergy and cancer (29, 51–53). In cancer settings, Gal-1 contributes to create immunosuppressive microenvironments, allowing tumor cell evasion of immune responses (46, 54–63). On the other hand, in experimental models of autoimmune disease including collagen-induced arthritis (64), myelin-oligodendrocyte glycoprotein_35–55_-induced encephalomyelitis (43, 65), diabetes (66), uveitis (67), and orchitis (68), Gal-1 elicits a broad spectrum of immunoregulatory activities leading to the resolution of chronic inflammation. The mechanisms underlying these immunosuppressive effects recapitulate those observed *in vitro* and *in vivo* including T-cell dysfunction and inhibition of proinflammatory cytokines (43, 58, 64, 69, 70), induction of tolerogenic DCs (40), expansion of Foxp3^+^ and Foxp3^−^ Tregs (60, 67) and generation of alternatively activated “M2-type” macrophages (71).

## Galectins in the Gut: A Sweet Path at the Cross-Roads of Tolerance and Inflammation

Despite the broad immunoregulatory activities of galectins, only few studies have uncovered the role of these lectins in gut immune homeostasis and the implications of these findings in intestinal inflammation. Interestingly, Gal-1, -2, -3, -4, and -9 are typically expressed in particular gut areas: whereas Gal-1 is mainly present in the lamina propria (LP), Gal-2, -3, -4, -7, and -9 are constitutively expressed within the epithelial compartment of the mouse intestine (72, 73). Epithelial cells (ECs) of small and large intestine express high levels of Gal-3 and Gal-4, although Gal-2 is only found in the large intestine (72). Interestingly, while Gal-3 may interact with commensal bacteria possibly influencing their colonization capacity (74), Gal-4 and Gal-8 mediate bacterial recognition and killing (75). Notably, Gal-1 is broadly expressed in small bowel enterocytes and may influence their viability (76). Moreover, studies reporting the galectin signature of human intestinal cells were mainly focused on pathologic conditions. Thus far, Gal-1, -3, -4, and -9 have shown to be homogeneously expressed across different sections of the large intestine (77).

## Galectins in Intestinal Inflammatory Diseases

### Inflammatory Bowel Diseases (IBD)

Crohn’s disease (CD) and ulcerative colitis (UC) represent the two main forms of IBD, chronic relapsing inflammatory conditions that affect the gastrointestinal tract. Despite some shared clinical features, these diseases can be distinguished by differences in risk factors, and clinical, anatomical, histological, and immunological features (78–80). Both conditions may involve an aberrant activation of mucosal T-cells against the commensal microbiota and deregulation of the EC compartment, thus compromising normal intestinal function and promoting an exuberant inflammatory response (81, 82). Whereas CD is characterized by an overactivation of mucosal Th1 and/or Th17 cells (with the concomitant secretion of IFN-γ, IL-17, and IL-22), UC patients exhibit a marked Th2 bias (with higher levels of IL-5 and IL-13) (79, 80, 83).

**Figure 1:**
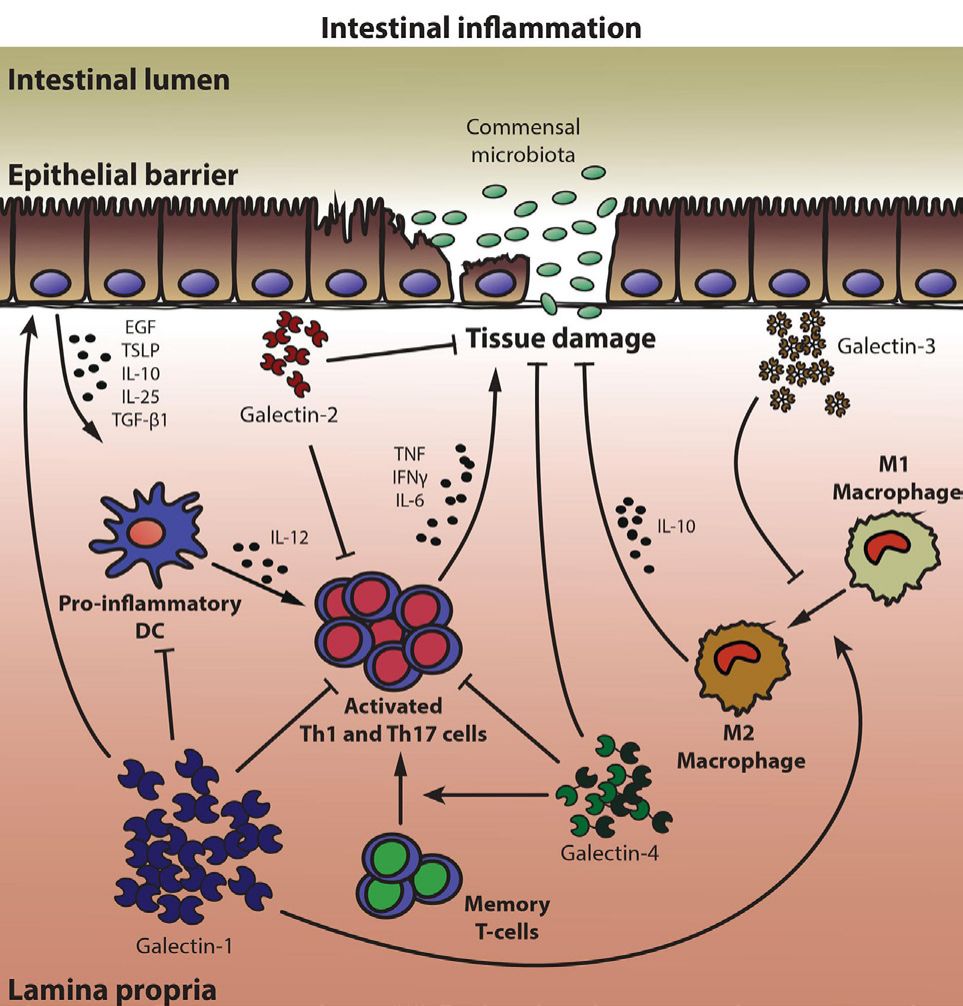
Multifunctional roles of galectins in mucosal intestinal inflammation. A coordinated network of galectin family members, which exerts either anti-inflammatory or proinflammatory responses, conditions epithelial barrier maintenance and immune gut homeostasis. Galectin-1 (Gal-1) promotes the secretion of growth factors and anti-inflammatory cytokines by epithelial cells (ECs), induces apoptosis of activated Th1 and Th17 cells, inhibits secretion of proinflammatory cytokines by dendritic cells (DCs) and T lymphocytes, and favors an anti-inflammatory (M2) macrophage phenotype. Similar to Gal-1, Gal-2 displays several anti-inflammatory properties, but also promotes wound healing and tissue regeneration in ECs. In animal models of colitis, Gal-3 shows mostly proinflammatory functions, inhibiting the polarization of macrophages toward an M2 phenotype, whereas Gal-4 exhibits both anti- and proinflammatory properties within the intestinal inflamed mucosa, depending on the experimental setting analyzed. By blocking production of proinflammatory cytokines, Gal-4 prevented inflammation and favored epithelial regeneration. However, through binding to memory T-cells, Gal-4 led to T-cell activation and perpetuated intestinal inflammation.

In a murine model of acute and chronic 2,4,6-trinitrobenzenesulfonic acid (TNBS)-induced colitis, treatment with recombinant Gal-1 (rGal-1) resulted in improvement of the clinical, histopathological, and immunological manifestations of the disease. Further analysis revealed increased apoptosis of TNBS-specific CD4^+^ T-cells in the LP, decreased percentage of activated T-cells and diminished levels of proinflammatory and Th1-type cytokines, effects that were accompanied by normalization of the mucosal architecture (69). Accordingly, Gal-1 was found to be upregulated in inflamed areas of IBD patients when compared with non-inflamed areas of the same patient or with control subjects. Indeed, expression of common mucosal-associated galectins (Gal-1, -3, -4, -9) was found dysregulated in these inflamed tissues, suggesting that alteration in galectin expression pattern may represent an endogenous compensatory mechanisms likely aimed at limiting the inflammatory process and restoring mucosal homeostasis (77). Notably, the viability of human and mouse enterocytes was also controlled by Gal-1 in human IBD biopsies and in murine models of intestinal inflammation. Interestingly, proinflammatory stimuli promoted Gal-1 binding to EC which in turn influenced their survival and secretion of proresolving cytokines, thereby protecting the intestinal epithelium from inflammatory responses (76, 84). Thus, through elimination of antigen-experienced T-cells, modulation of proinflammatory cytokines or direct stimulation of epithelial-derived anti-inflammatory factors, Gal-1 contributes to the resolution of gut inflammation (Figure 1).

Notably, other members of the galectin family could also be involved in controlling intestinal inflammation (Figure 1). Gal-3 may function as a proinflammatory mediator that aggravates dextran sulfate sodium (DSS)-induced colitis through promotion of an M1 macrophage phenotype (85). Deletion of Gal-3 gene in mice or pharmacological inhibition of this lectin promoted macrophage polarization toward a M2 phenotype in colonic tissue (85). In line with these observations, peritoneal macrophages lacking Gal-3 are more prone to undergo apoptosis than their wild-type counterparts, strongly suggesting a role for Gal-3 as a proinflammatory mediator in the peritoneal cavity (86). Notably, in IBD patients Gal-3 levels are reduced in active inflamed areas, probably aimed at limiting the inflammatory process and restoring mucosal homeostasis (77, 87–89). In contrast, a protective role for this lectin was suggested in both the DSS-induced and the T-cell transfer colitis models, through suppression of IL-6 production by colonic LP fibroblasts or by induction of Foxp3^+^ Tregs (90, 91). These discrepancies could be explained not only by differences in experimental models (92), but also by dissimilar roles of endogenous versus exogenous Gal-3 during different stages of the inflammatory response (16, 29).

Similarly, Gal-4 has been shown to act either as an anti-inflammatory or as a proinflammatory factor in IBD. An anti-inflammatory function for both Gal-4 and Gal-2 was described, which contributed to ameliorate mucosal inflammation in the DSS colitis model through mechanisms involving apoptosis of activated mucosal LP T-cells and diminished proinflammatory cytokine secretion (93, 94) (Figure 1). Within the EC compartment, Gal-2 and Gal-4 (but not Gal-1) promoted wound-healing (95). Gal-4 may also function as a glycoprotein trafficking carrier, which generates an apical endocytic-recycling pathway *via* complex-type N-glycans (96, 97). Notably, during IBD progression, local inflammation was also associated with dysregulated expression of glycosyltransferases, leading to exposure of altered glycan structures on memory CD4^+^ T-cells (98). In fact, downregulation of core 2 β1,6-*N*-acetylglucosaminyltransferase 1 (C2GnT1) allowed Gal-4-O-glycan interactions resulting in expansion of memory CD4^+^ T-cells, enhanced IL-6 production and perpetuation of intestinal inflammation (98, 99). Notably, inflamed IBD mucosa could be distinguished from control tissue and from other types of intestinal inflammatory conditions by a specific galectin signature, as revealed by a multivariate-linear discriminant analysis of Gal-1, -3, -4, and -9 in IBD patient biopsies (77).

### Celiac Disease

Oral tolerance to dietary antigens is a key active process in which immune responses to innocuous antigens, commensal bacteria, and pathogens are suppressed (100). In CeD, intolerance to indigestible wheat gluten peptides results in chronic intestinal inflammation associated with an extensive Th1 and Th17 responses (101). Similar to most chronic inflammatory diseases, CeD has a multifactorial etiology involving environmental factors as well as genetic components. Among them, HLA-DQ2 and HLA-DQ8 have been identified to confer susceptibility to CeD development (102–104). In genetically susceptible individuals, intestinal inflammation is triggered when ingested gliadin (proline-rich and glutamine-rich gluten proteins) found in wheat, rye, barley, and oats (105, 106) is partially processed and presented to CD4^+^ T-cells that infiltrate the LP of the small intestine. Thus HLA-DQ2/8 molecules may orchestrate a gluten-specific CD4^+^ T-cell response (107).

Celiac disease patients on a gluten-containing diet show increased levels of serum antibodies specific for gliadin and tissue transglutaminase, an enzyme that plays a key role in disruption of tolerance to gluten, among other antigens (108, 109). To date, the only known effective treatment for CeD is a lifelong gluten-free diet (GFD) (109), which allows the complete recovery of intestinal structure and function, and normalization of serum antibodies (110). In spite of considerable progress in our understanding of the mechanisms underlying CeD development and progression, there is no clear answer to how breaking mucosal tolerance to gluten turns a controlled local immune response into chronic inflammation and epithelial destruction (111).

Although the involvement of galectins in IBD has been well documented, their relevance in CeD development and progression is poorly understood. In this regard, a significant increase in Gal-10 expression has been correlated with mucosal damage and number of eosinophils in duodenal lesions of CeD patients (112). In addition, despite some discrepancies, evidence suggest a role for Gal-9 in human and mouse food allergy, a broad entity with some common features with CeD (113–115).

## Galectin-1 Expression Delineates Response to GFD in CeD Patients

Since several immunoregulatory mechanisms are dysregulated in mucosal tissue of CeD patients (108) and Gal-1 displays broad tolerogenic and anti-inflammatory activities in mucosal tissues (29), we evaluated the expression of this lectin in biopsies of CeD patients with or without gluten withdrawal (Table 1).

**Table 1:** Analysis of duodenal biopsies from control subjects, untreated CeD patients, and CeD patients subjected to gluten withdrawal.

Characteristics	CeD	CeD-GFD
Number of cases and gender (female/male)	10 (8/2)	10 (7/3)
Median age, years (range)	32 (18–56)	37 (24–67)
Median time on a GFD, years (range)	–	4 (2–14)

**Number of cases with positive serology**		
IgA tissue transglutaminase > 20 UA/mL	10	3

**Severity of histologic damage**		
(Marsh 3 classification) number of patients	10	2

*Demography, histological, and serology data of untreated (at diagnosis) celiac disease patients (CeD) and of CeD treated with gluten withdrawal (CeD-GFD) whose duodenal biopsies were employed for the study*.

Patients were diagnosed with CeD according to conventional clinical, serological and histological criteria (108, 117). Control subjects: non-celiac subjects with negative CeD serology and normal duodenal histology, *n* = 10. Patients and controls were informed in detail about the study, and written consent was obtained. The protocols were approved by Ethics Committees of Hospital “Carlos B. Udaondo.”

**Figure 2:**
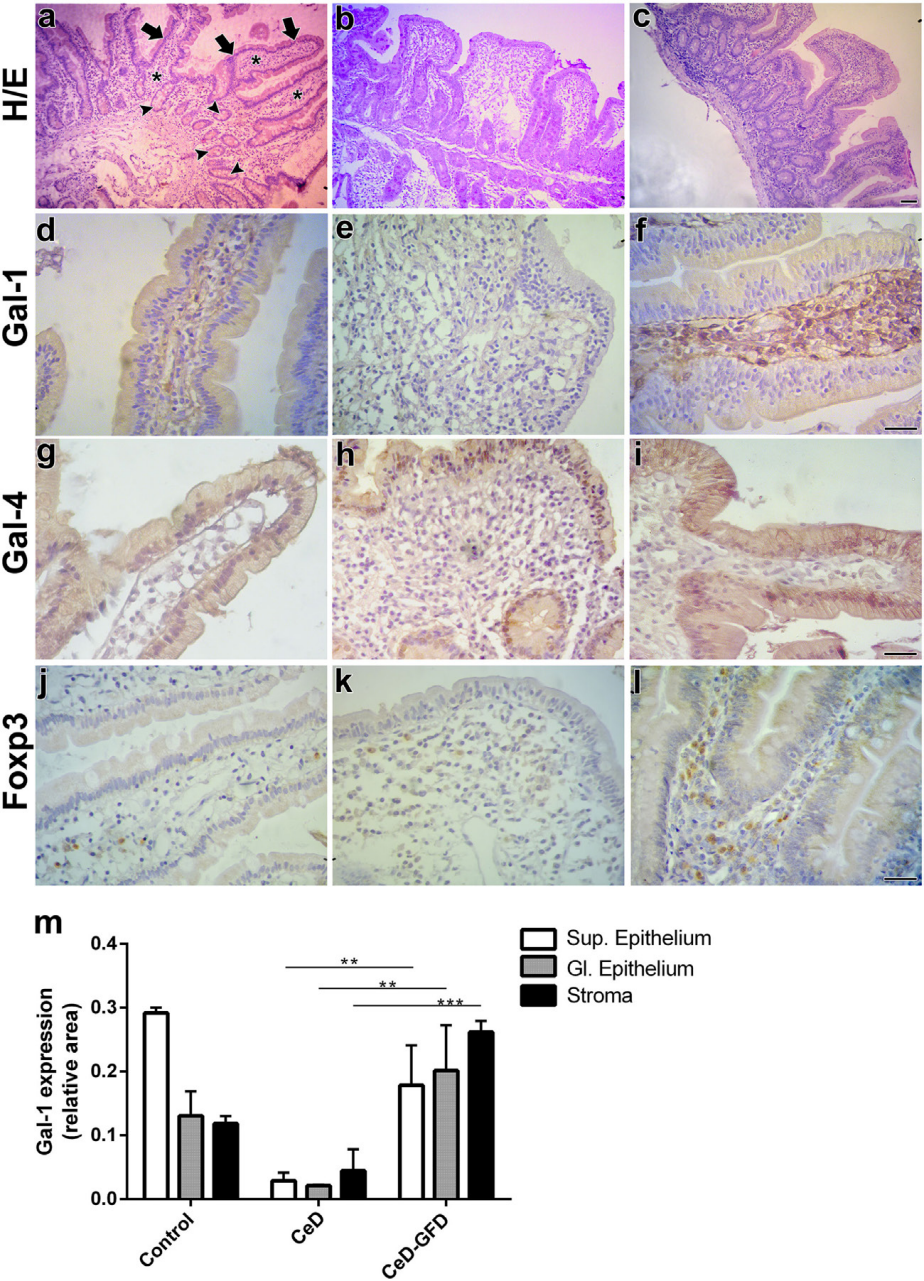
Expression of galectin-1 (Gal-1), Gal-4, and Foxp3 in response to gluten-free diet (GFD) in duodenal biopsies from celiac disease (CeD) patients. Representative micrographs of control subjects, untreated CeD patients and CeD patients subjected to gluten withdrawal (CeD-GFD patients) are shown. **(A–C)** Hematoxylin/eosin (H/E) staining of paraffin-embedded sections of duodenal biopsies from **(A)** control subjects, **(B)** CeD patients, and **(C)** CeD-GFD patients. Arrows indicate the superficial epithelium, and arrowheads indicate the glandular epithelium while asterisks denote the stroma. Bar = 20 µm. **(D–F)** Immunohistochemical analysis of Gal-1 expression in duodenal biopsies from control subjects **(D)**, CeD patients **(E)**, and CeD-GFD patients **(F)**. Bar = 20 µm. **(G–I)** Immunohistochemical analysis of Gal-4 expression in duodenal biopsies from control subjects **(G)**, CeD patients **(H)**, and CeD-GFD patients **(I)**. Bar = 20 µm. **(J–L)** Immunohistochemical analysis of Foxp3^+^ cells in biopsies from control subjects **(J)**, CeD patients **(K)**, and CeD-GFD patients **(L)**. Bar = 20 µm. **(M)** Quantification of Gal-1 expression determined by immunohistochemistry. Bars represent immunostained area corresponding to superficial (Sup) and glandular (Gl) epithelium, and stroma, in paraffin sections from duodenal biopsies from controls, untreated CeD patients and CeD-GFD patients. Evaluation of staining intensity was performed with the Image J software (NIH, Bethesda, MD, USA). One-way ANOVA Tukey test was used for multiple comparisons. **p < 0.01, ***p < 0.001.

Hematoxylin/eosin staining of duodenal biopsies showed that, unlike the conserved LP structures observed in control subjects (Figure 2A), CeD patients exhibited atrophic villi with enlarged hyperplastic crypts and increased intraepithelial lymphocytes infiltration (Figure 2B). Mucosa from CeD patients after GFD (CeD-GFD patients) presented considerably recovered villi (Figure 2C). In control biopsies, Gal-1 labeling (Table 2) was mainly localized in stromal cells, while most ECs exhibited weak positive staining (Figure 2D). Biopsies from CeD patients exhibited a poorly labeled stromal fibrillar network, while atrophic epithelia showed no considerable staining. Subepithelial and periglandular infiltrating cells appeared negative for Gal-1 (Figure 2E). Duodenal biopsies from CeD-GFD patients exhibited a substantial increase in Gal-1 immunoreactivity, especially in the interstitium of the recovered villi. Numerous subepithelial fibroblast-like cells, as well as round nucleus-containing cells scattered in the LP compatible with macrophages, and a few lymphocytes were Gal-1-positive. Notably, ECs recovered their Gal-1 weak positive staining (Figure 2F). Moreover, no significant differences were observed in the expression of Gal-4 (Table 2)—a galectin family member mostly expressed in ECs of the intestinal tract—in biopsies from CeD patients before or after gluten withdrawal (Figures 2G–I).

**Table 2:** Analysis of duodenal biopsies from control subjects, untreated CeD patients, and CeD patients subjected to gluten withdrawal.

Antigen	Primary antibody	Secondary antibody
Gal-1	In-house rabbit anti-Gal1 antibody (1:500) (64)	anti-rabbit biotinylated antibody (1:130) (Amersham Pharmacia, Buckinghamshire, UK)
Gal-4	Goat anti-Gal-4 antibody (1:75) (Santa Cruz Biotech, Dallas, TX, USA)	anti-goat biotinylated antibody (1:180) (Amersham Pharmacia, Buckinghamshire, UK)
Foxp3	Rabbit anti-Foxp3 antibody (1:50) (Abcam, Cambridge, UK)	anti-rabbit biotinylated antibody (1:130) (Amersham Pharmacia, Buckinghamshire, UK)

*Antibodies used for immunohistochemical analysis of Gal-1, Gal-4, and Foxp3 expression in duodenal biopsies from control subjects and patients*.

*Four intestinal biopsies from the second duodenal section from each patient and control were collected*.

Overall, while control duodenal biopsies showed moderate Gal-1 staining, and both epithelium and stroma from untreated CeD patients were poorly labeled, CeD-GFD biopsies showed a dramatic increase in Gal-1 immunoreactivity (p < 0.001; Figure 2M), which correlated with normalization of duodenal mucosal structure. Interestingly, the expression of stromal Gal-1 in these patients was not only recovered but also increased in intensity compared with control biopsies (Figures 2D–F,M).

To further characterize the underlying inflammatory response and given the association of Gal-1 with induction of Foxp3^+^ Tregs, we analyzed the expression of this transcription factor in inflammatory infiltrates (Table 2). Though less accurate in defining human Tregs than mouse Tregs (116), determination of Foxp3 staining is typically considered a reliable indicator of the suppressive tissue microenvironment. An increased number of Foxp3^+^ cells was observed in CeD-GFD patients (Figures 2J–L), which positively correlated with Gal-1 expression, suggesting activation of a circuit of immunosuppressive events leading to restoration of mucosal homeostasis. Further studies should be aimed at addressing the immunosuppressive potential of this tolerogenic circuit in functional assays.

Our findings suggest that, in response to gluten withdrawal, upregulation of Gal-1 might contribute to restrain the chronic inflammatory response, thus allowing the onset of the recovery process leading to remission of mucosal damage and reestablishment of villi structure. In addition, decreased Gal-1 expression observed in untreated CeD patients compared to control individuals may suggest a role for this lectin in controlling gut homeostasis under physiologic conditions. Interestingly, modulation of Gal-1 expression during CeD development appeared to be specific as no differences were found in the expression of Gal-4, suggesting selective regulation of individual galectins during mucosal inflammation.

## Conclusion

The delicate balance between host immunity and tolerance allows the maintenance of gut homeostasis avoiding detrimental intestinal inflammation. Data presented here, resulting both from published information (Figure 1) and new observations (Figure 2), highlight the role of galectins as active players of complex regulatory circuits operating in intestinal mucosal tissue to preserve immune and epithelial homeostasis. While galectins (particularly Gal-1, -2, -3, -4, and -9) may be critical in preserving intestinal homeostasis, an initial set up in which galectins’ expression is altered or the intestinal glycome is reprogrammed may influence development of intestinal inflammation.

To gain insight into the role of Gal-1 in CeD patients, we demonstrated here an increase in Gal-1 expression following GFD that was accompanied by an increased frequency of Foxp3^+^ cells. The coordinated action of both immunosuppressive mechanisms may occur as synchronized events to generate a tolerogenic milieu in mucosal tissue of treated patients. Since tolerance to gluten peptides would be hard to reestablish under sustained inflammatory conditions, the antigen challenge-free time window (achieved by gluten withdrawal) may allow the development of these immunosuppressive pathways. The subsequent resolution of the inflammatory response may foster the onset of the recovery process, leading to remission of mucosal damage and reestablishment of villi structures.

In line with findings observed in other intestinal inflammatory conditions (76, 77, 84), our observations support the use of Gal-1 agonists to treat severe mucosal inflammation. In addition, Gal-1 may serve as a potential biomarker to follow up CeD progression.

Challenges for the future will embrace the rational manipulation of the Gal-1-glycan axis toward attenuating immune responses in CeD. Studies in *Lgals1*^−^*^/^*^−^ mice will be necessary to determine a putative role of Gal-1 and its specific ligands in supporting mucosal tolerance to gluten. Moreover, the ability of rGal-1 to suppress intestinal inflammation should also be evaluated in experimental CeD models. In this regard, evidence stemming from the study of experimental models of autoimmunity, chronic inflammation, fetomaternal tolerance, and tumor growth provides fundamental insights into the critical role of this lectin and its specific glycosylated ligands in maintaining and restoring immune tolerance and homeostasis, thus encouraging future implementation of Gal-1-based therapies in CeD patients.

## Ethics Statement

Patients and controls were informed in detail about the study, and written consent was obtained. The protocols were approved by Ethics Committees of Hospital “Carlos B. Udaondo.”

## Author Contributions

VS acquired data, analyzed and interpreted data, and wrote the manuscript. AQ developed methodology, analyzed and interpreted data, and revised the manuscript. LM analyzed and interpreted data and revised the manuscript. SN analyzed data, managed patients, and revised the manuscript. AC and ES, and EM managed patients and revised the manuscript. KM analyzed and interpreted data and wrote the manuscript. JB and CM conceived and designed the study, analyzed and interpreted data, and revised the manuscript. GR conceived and designed the study, analyzed and interpreted data, and wrote the manuscript.

## Conflict of Interest Statement

The authors declare that the research was conducted in the absence of any commercial or financial relationships that could be construed as a potential conflict of interest.
